# Unraveling ADHD for the pediatrician

**DOI:** 10.1016/j.jped.2025.101464

**Published:** 2025-11-01

**Authors:** Erasmo Barbante Casella, Beatriz Borba Casella

**Affiliations:** Instituto da Criança do Hospital das Clínicas da Faculdade de Medicina da Universidade de São Paulo, São Paulo, SP, Brazil

**Keywords:** Attention-deficit disorder with hyperactivity, Pediatrics, Diagnosis, Therapeutics, Primary health care

## Abstract

**Objective:**

To review current evidence on the pathophysiology, clinical features, differential diagnosis, and therapeutic management of Attention-Deficit/Hyperactivity Disorder (ADHD), emphasizing the pediatrician’s role in diagnostic recognition and coordination of multidisciplinary care.

**Data sources:**

Narrative synthesis of the contemporary literature addressing genetic and neurobiological underpinnings, diagnostic criteria, comorbidities, and treatment strategies relevant to pediatric practice.

**Summary of findings:**

ADHD is a multifactorial neurodevelopmental disorder involving genetic predisposition and neurobiological alterations, particularly in cortico-subcortical circuits related to dopaminergic and noradrenergic modulation. Clinical presentation varies with age and context and may be accompanied by comorbidities such as learning disorders, anxiety, and depression. Diagnosis is clinical, based on standardized criteria and assessment across multiple settings, and requires exclusion of conditions that mimic ADHD. Management is multimodal, encompassing psychosocial interventions, school-based support, and, when indicated, pharmacological therapy. Pediatricians serve as the first point of contact and play a central role in early identification, family guidance, and integration of healthcare and educational teams.

**Conclusion:**

Proactive pediatrician involvement is essential for early diagnosis and effective management of ADHD, supporting better prognosis and the child’s overall development.

## Introduction

Attention-Deficit/Hyperactivity Disorder (ADHD) is one of the most common neurodevelopmental disorders in childhood, with a global prevalence estimated between 5 % and 7 %, varying according to diagnostic criteria and sociocultural context [1]. It is characterized by persistent patterns of inattention, hyperactivity, and/or impulsivity that interfere with academic performance, social relationships, and emotional well-being.

The pediatrician holds a pivotal responsibility in the identification of ADHD, often serving as the first referral source for families and educational institutions when behavioral concerns or academic underperformance arise. This role demands a current understanding of the disorder’s pathophysiology, clinical manifestations, differential diagnosis, and evidence-based therapeutic strategies, as well as the capacity to coordinate a multidisciplinary, child- and family-centered approach.

## Epidemiology

Epidemiological data highlight the global impact and persistence of ADHD across the lifespan. Polanczyk et al. (2007), in a comprehensive meta-analysis, observed a global prevalence of ADHD of 5.29 % among individuals aged six to 17 years (95 % confidence interval: 5.0–5.6) [2]. With brain maturation, some patients experience significant clinical improvement; however, approximately 60–70 % of individuals remain symptomatic into adulthood [3].

Among children and adolescents, the male-to-female ratio in ADHD is approximately 2.4:1 in the general population². However, this ratio is considerably higher (around 4:1) among those seeking treatment, likely due to the greater externalizing and disruptive behaviors observed in boys, which make them more readily identified and referred for clinical evaluation [4,5]. As individuals age, this sex ratio tends to decrease to 2:1 during adolescence, and to between 1.9:1 and 1.2:1 in adulthood based on registry and claims data, and as low as 1.1:1 in population-based surveys [6,7]. These shifting sex ratios across the lifespan are attributed to multiple factors, including delayed recognition and diagnosis of ADHD in females, an increasing number of adult women seeking help, and the reduced diagnostic emphasis on hyperactivity in adulthood, an ADHD symptom domain that is less prevalent in females [8]. Additionally, while referral during childhood often depends on parents or teachers, in adulthood, self-referral becomes more common. Women are more likely than men to seek professional assistance for mental health concerns [9].

## Etiology and pathophysiology

The exact mechanisms underlying the pathogenesis of ADHD are not yet fully elucidated. Current research suggests that ADHD is a complex, multifactorial disorder, with multiple genetic, environmental, and neurobiological factors contributing to its development.

A variety of factors have been identified as potential contributors to the etiology of ADHD, including prenatal exposure to substances such as alcohol, cocaine, and lead, as well as perinatal factors such as prematurity, hypoxia, and maternal stress [10]. Additionally, early brain injuries and adverse childhood experiences (ACEs) are thought to play a role in the development and phenotypic expression of the disorder [10]. Further evidence links ADHD with early-life traumatic brain injury, as well as nutritional and emotional deprivation [11].

Family, twin, and adoption studies, along with molecular investigations, consistently highlight genetics as the primary determinant in the etiology of ADHD. Heritability estimates suggest a strong genetic component, with studies indicating that the likelihood of an identical twin also developing ADHD is approximately 76 % [12]. Meta-analyses have further elucidated the involvement of multiple genetic variants in ADHD, although their individual effects are modest. These genetic factors are believed to interact with environmental influences, contributing to the disorder’s complex etiology [13].

The largest genome-wide association studies (GWAS) of ADHD have identified 27 significant loci across the genome, implicating 76 genes, many of which are upregulated during early brain development [14]. The genetic risk for ADHD is particularly enriched in genes associated with specific neuronal subtypes, including midbrain dopaminergic neurons. Notably, genes such as FOXP1 and FOXP2, which are linked to speech disorders and intellectual disabilities, as well as SORCS3, PTPRF, and MEF2C, which are involved in synaptic connectivity, have been implicated in ADHD pathogenesis [14]. Despite these promising findings, it is important to note that genetic testing remains neither sufficiently accurate nor recommended for the diagnosis of ADHD or for guiding pharmacological treatment [13,15].

Molecular genetic studies indicate a polygenic contribution to ADHD risk, with the heritability of common genetic variants (single nucleotide polymorphisms, SNPs) estimated to range from 14 % to 20 % [14]. Furthermore, genetic variants associated with ADHD have been linked to alterations in brain structure, including smaller brain volumes and disrupted functional connectivity, particularly in subcortical regions [16].

While the complete biochemical mechanisms underlying ADHD remain unclear, pharmacological, neuroimaging, and lesion studies provide compelling evidence that catecholamines, dopamine, and norepinephrine are central to the disorder's pathophysiology [17]. Other neurotransmitters, including acetylcholine (ACh), serotonin, and glutamate, have also been implicated in modulating various cognitive processes, with ACh playing a particularly significant role in short- and long-term memory, as well as attentional processing, especially in the domains of stimulus detection and response selection [17].

Neuropsychological and neuroimaging studies consistently highlight impairments in prefrontal cortex functions in individuals with ADHD, underscoring the critical role of this region in the expression of the disorder's symptoms. Shaw et al., in their investigation of cortical development trajectories in ADHD, demonstrated delayed maturation across several brain regions, particularly within prefrontal areas [18]. Multiple studies emphasize the significance of alterations in neural networks that connect these regions [19].

These brain changes suggest that, in addition to core symptoms such as hyperactivity, impulsivity, and inattention, ADHD patients exhibit deficits in higher-order executive functions, including perception, planning, organization, and inhibition of behavior, motor responses, and thought processes [20].

Neuroimaging studies further suggest that ADHD is associated with both structural immaturity and atypical functional development of the brain. Functional and structural neuroimaging findings reveal alterations in key regions such as the prefrontal cortex, cingulate gyrus, basal ganglia, corpus callosum, cerebellum, fusiform gyrus, and parietal and temporal lobes. These regions are involved in the regulation of attention, behavioral inhibition, and motor control—functions that rely on complex interactions among dopaminergic, noradrenergic, serotonergic, glutamatergic, and cholinergic projections [14,19]. Taken together, this evidence highlights cortical maturation delays and dysfunctions within cortico-subcortical networks that modulate executive functions [20].

Imaging studies have consistently revealed reduced volumes in the basal ganglia and limbic system, along with thinner cortical areas in the ventromedial orbitofrontal regions. Additionally, these studies have shown decreased integrity in neural tracts involved in visual attention, including the posterior corpus callosum (which connects temporo-parieto-occipital regions), the sagittal striatum, and the left inferior longitudinal and uncinate fasciculi [20].

Functional MRI studies investigating cognitive control in ADHD consistently reveal underactivation of the inferior frontal cortex and insula, as well as reduced activation in the right or bilateral dorsolateral prefrontal cortex during attention and working memory tasks [20].

Research on the Default Mode Network (DMN), a collection of brain regions that are active during rest and internally focused states, such as mind-wandering and self-referential thoughts, has shown alterations in ADHD. The DMN includes the posterior cingulate cortex, medial prefrontal cortex, inferior parietal lobule, lateral temporal cortex, middle temporal/angular gyrus, and the superior frontal gyrus/rostral anterior cingulate cortex. This network is typically engaged in recalling past events, envisioning future scenarios, and processing internal thoughts. Some researchers suggest that individuals with ADHD struggle because their DMN remains active even during tasks requiring focused attention, which disrupts cognitive control. In contrast, neurotypical individuals can deactivate their DMN when concentration is needed, allowing for better attentional focus [21].

Longitudinal MRI studies provide evidence of delayed maturation in children with ADHD, particularly in terms of cortical thickness and surface area, with the greatest delays observed in frontal and temporal regions, as well as in cerebellar white matter [22]. Moreover, studies show delayed functional maturation in the resting-state DMN, cognitive control networks, and ventral attention systems, alongside reduced anticorrelation between the DMN and these networks. Notably, diminished connectivity with the right inferior prefrontal cortex has been linked to greater inattention in later stages of life [22].

## Diagnosis

The diagnosis of ADHD is primarily clinical, based on the assessment of symptoms and behavioral signs across two or more contexts (e.g., home, school, social activities). The use of validated scales (e.g., SNAP-IV) can help structure the evaluation, but they should not be relied upon solely for diagnostic purposes.

The diagnosis should follow the criteria established by the DSM (DSM-5-TR) or the ICD (ICD-11). Although they present minor differences, both emphasize the need to examine the manifestation of symptoms across various contexts and levels of stimulation. In Brazil, the DSM-5-TRis is generally preferred.

According to DSM-5-TR, symptoms are categorized into two domains:

• Inattention: difficulty maintaining focus, disorganization, careless mistakes, and avoidance of tasks that require prolonged attention.
• Hyperactivity/Impulsivity: motor restlessness, excessive talking, frequent interruptions, and difficulty waiting one's turn.

The clinical presentation may be predominantly inattentive, predominantly hyperactive/impulsive, or combined. In young children, hyperactivity is often more prominent, while in adolescents and young adults, inattention tends to be more apparent.

DSM-5-TR presents a list of 18 symptoms, nine related to inattention and nine to hyperactivity/impulsivity (Table 1).

**Table 1:** Diagnostic criteria for ADHD (DSM-5 TR).

**A. Six (or more) of the following inattention symptoms (minimum duration of 6 months):** a) Fails to pay close attention to details or makes careless mistakes in schoolwork, work, or other activities. b) Has difficulty sustaining attention in tasks or play activities. c) Often does not seem to listen when spoken to directly. d) Does not follow through on instructions and fails to finish schoolwork, chores, or duties in the workplace. e) Has difficulty organizing tasks and activities. f) Avoids or is reluctant to engage in tasks requiring sustained mental effort. g) Frequently loses items necessary for tasks or activities. h) Is easily distracted by extraneous stimuli. i) Is often forgetful in daily activities. **B. Six (or more) of the following hyperactivity/impulsivity symptoms (minimum duration of 6 months):** a) Fidgets with hands or feet or squirms in seat. b) Leaves the seat in situations where remaining seated is expected. c) Runs about or climbs excessively in inappropriate situations. d) Has difficulty playing or engaging quietly in leisure activities. e) Is often “on the go” or acts as if “driven by a motor.” f) Talks excessively. g) Blurts out answers before a question has been completed. h) Has difficulty waiting for their turn. i) Interrupts or intrudes on others.

For the diagnosis in patients up to 17 years old, the predominantly hyperactive/impulsive presentation requires six (or more) of the nine DSM-5 hyperactivity criteria during the past six months [23]. The same applies to the predominantly inattentive presentation [23]. For a combined presentation, both inattention and hyperactivity criteria must be met [23]. For individuals aged 17 and older, the threshold is reduced to five (or more) symptoms in either the inattentive or hyperactive/impulsive category, reflecting developmental changes in symptom presentation over time.

It is important to emphasize that several of these symptoms may also be present in children with typical development. Therefore, for an ADHD diagnosis, symptoms must be frequent, occur in at least two different settings, and cause impairments in social, academic, or occupational functioning [23]. Thus, it is essential to gather information about the child’s behavior not only at home but also at school, in sports, and in other activities.

Different from previous editions, DSM-5 now allows the coexistence of an ADHD diagnosis with autism and requires that symptoms be present before the age of 12 [23].

The use of ancillary tests is generally not indicated for the diagnosis of ADHD, but may be considered in atypical cases or when diagnostic uncertainty exists. For example, an electroencephalogram to rule out absence seizures, or imaging to exclude structural brain abnormalities. Neuropsychological assessment is not required for routine ADHD diagnosis, but it can be very helpful in planning patient management.

## Differential diagnosis and comorbidities

Several conditions can either mimic or occur concomitantly with ADHD, exacerbating its symptoms. In fact, in 60 % to 70 % of cases, ADHD is comorbid with another disorder, which can intensify the impairments associated with ADHD. When these comorbid conditions are not identified and treated, they can significantly hinder the effectiveness of treatment [5].

A detailed history, gathering information from school, and assessment of neuropsychomotor development are essential for diagnostic accuracy.

The most common comorbidities associated with ADHD include anxiety disorders, oppositional defiant disorder, conduct disorder, autism spectrum disorder, bipolar affective disorder, depression, and specific learning disorders [5].

Additionally, many of the conditions listed as potential comorbidities may also occur independently of ADHD, which can contribute to misdiagnosis. The most common of these include anxiety disorders, depression, autism spectrum disorder, oppositional defiant disorder, sleep disorders, dyslexia, and dyscalculia [5].

Moreover, other conditions may mimic ADHD and must be ruled out during the diagnostic process, such as auditory or visual impairments, sleep disturbances, certain types of epilepsy, endocrinological disorders like hypothyroidism or adrenal insufficiency, severe anemia, motivational deficits, or even ineffective educational practices [5].

The authors emphasize the need for great rigor in the diagnosis of ADHD, which, in the absence of biological markers, requires a very detailed history and physical examination, as well as investigation of the child’s behavior in other settings, either directly or indirectly. This can be carried out through questionnaires such as the SNAP-IV, which is freely available and widely used in the context.

## Management

### Pharmacological treatments

Most international guidelines consider pharmacotherapy as the primary treatment option for ADHD, although the American Academy of Pediatrics initially recommends combining medication with behavioral therapy [24].

The medications approved for ADHD by the FDA and other regulatory agencies are stimulants (methylphenidate and amphetamine-based formulations) and non-stimulants (atomoxetine, and α2-adrenergic agonists such as extended-release (ER) clonidine and guanfacine). Stimulants and atomoxetine are approved for both children and adults, while extended-release clonidine and guanfacine are approved in the United States only for children, though studies have shown guanfacine ER to be effective in adults as well [25].

Numerous randomized controlled trials have consistently demonstrated the efficacy of pharmacological treatments in reducing ADHD symptoms [26]. Clinical guidelines recommend stimulants as the first-choice medication due to their robust effectiveness. According to NICE, methylphenidate is the initial treatment of choice for children; if response is inadequate or adverse effects occur, lisdexamfetamine should be considered, and subsequently non-stimulants may be used. In adults, either amphetamines or methylphenidate may be initiated as first-line options, with non-stimulants reserved for later stages [27]. In contrast, North American guidelines do not establish a preference between methylphenidate and amphetamines for children or adults. Methylphenidate exerts its effect primarily by inhibiting dopamine and norepinephrine reuptake at presynaptic neurons. Amphetamine derivatives, such as lisdexamfetamine, share this mechanism but additionally enhance the release of these catecholamines from presynaptic vesicles. Beyond pharmacotherapy, multimodal interventions remain essential, encompassing psychoeducation of families, school-based strategies, and cognitive and/or behavioral therapy delivered by multidisciplinary teams [5].

The treatment of ADHD should always be guided by a comprehensive diagnostic process, including the identification of each patient’s strengths and vulnerabilities, followed by the establishment of a treatment plan with clearly defined goals to be reassessed at every follow-up.

Providing families and patients with accurate information about the neurobiological and genetic basis of ADHD is crucial to fostering understanding of the challenges faced and promoting adherence to therapeutic strategies.

According to the FDA, pharmacological treatment is formally approved for use from six years of age. However, the American Academy of Pediatrics notes that, in specific circumstances, medication may be considered from the age of four, strictly when psychoeducation, behavioral interventions, and environmental adjustments prove insufficient [24].

Table 2 presents the different medications currently available in Brazil for ADHD treatment, together with their pharmacokinetic characteristics. Dosages are relatively variable, depending on symptom severity and individual tolerability to side effects***.***

**Table 2:** Pharmacokinetics of the main medications that can be used in ADHD therapy.

Medication	Presentations	Half-life (hours)	Onset of action	Effect size
Immediate-release methylphenidate	10 mg	4	20–30 min	0.8–1.3
Methylphenidate LA (SODAS)	20 mg, 30 mg, 40 mg	8	30–120 min	0.8–1.3
Methylphenidate OROS	18 mg, 36 mg, 54 mg	12	30–120 min	0.8–1.3
Lisdexamfetamine	30 mg, 50 mg, 70 mg	13	120 min	0.8–1.5
Atomoxetine	10 mg, 18 mg, 25 mg, 40 mg, 60 mg, 80 mg, 100 mg	24	2–6 weeks	0.7
Imipramine	10 mg, 25 mg	6–18	2–4 weeks*	0.6
Bupropion	150 mg	3–4	1–2 weeks*	0.6
Clonidine	0.1 mg	6	30–60 min	0.58

⁎ Refers to the time required to achieve full treatment efficacy after reaching the desired dose. The main side effects of the medications used in the treatment of ADHD, which are generally well tolerated, are listed in Table 3.

Stimulants (methylphenidate or amphetamines) represent the first-line pharmacological treatment, with an estimated efficacy of approximately 85 %. The choice of pharmacological agents should consider factors such as the desired duration of action, the feasibility of multiple daily dosing, and financial constraints, given that long-acting formulations are significantly more expensive. If one stimulant proves ineffective or poorly tolerated, switching to the alternative stimulant is recommended before moving to non-stimulant options [24]. Short-acting methylphenidate (MPH), once absorbed, undergoes extensive hepatic metabolism, with approximately 80 % converted to ritalinic acid and rapidly excreted. The remaining fraction is metabolized into inactive derivatives, resulting in a short half-life of 2–3 hours. This pharmacokinetic profile requires administration two to three times daily, with gradual titration. The usual dose ranges from 0.3 to 0.5 mg/kg/dose, not exceeding 1 mg/kg/day or 60 mg/day [28].

In Brazil, MPH is also available in two long-acting formulations, which employ distinct release mechanisms: the Spheroidal Oral Drug Absorption System (SODAS) and the Osmotic Release Oral System (OROS).

• OROS formulation: Encapsulated insoluble system, where 22 % of the total dose is delivered immediately for rapid onset, while the remaining 78 % is released gradually via an osmotic pump mechanism. This design ensures approximately 12 hours of therapeutic effect, with peak plasma concentration achieved between 6 and 7 hours [28].
• SODAS formulation: Capsule containing microgranules of two sizes, providing 50 % immediate release and 50 % delayed release after ∼4 hours, mimicking two consecutive short-acting doses. The capsule can be opened and sprinkled on applesauce, which facilitates administration in children unable to swallow pills [28].

Although both formulations deliver bioequivalent doses of MPH, their distinct pharmacokinetic profiles may lead to interindividual variability in therapeutic response. Amphetamines represent another major class of stimulants for ADHD. In Brazil, the available option is lisdexamfetamine**,** a prodrug enzymatically converted in the bloodstream to its active metabolite**,** d-amphetamine. Randomized controlled trials demonstrate comparable efficacy and safety to MPH, with a longer duration of action (up to ∼13 hours [29]. The pharmacokinetics of d-amphetamine following lisdexamfetamine administration are monophasic and dose-proportional**,** reaching peak plasma concentration at approximately 2 hours, with a half-life of 8.6–8.9 hours [29].

In addition to stimulant therapy, non-stimulant medications such as atomoxetine, imipramine, alpha-adrenergic agonists (e.g., clonidine), and bupropion also play a role in the treatment of ADHD.

Since early 2023, atomoxetine has also become available in Brazil. Atomoxetine is a non-stimulant medication whose mechanism of action differs from stimulants because it does not target the nucleus accumbens directly. Its effect size is slightly smaller than that of stimulants (approximately 0,7), but it provides sustained action for about 24 hours, allowing for administration once or twice daily. Clinical benefits may begin to emerge after the second week of treatment, although the maximum therapeutic effect is generally observed after four to five weeks of continuous use [30].

Imipramine also has an established role in ADHD therapy. However, because its effect size is smaller than that of stimulants and it carries the risk of more serious adverse effects, clinical guidelines classify tricyclic antidepressants as third-line pharmacological options. An electrocardiogram (ECG) should be obtained before and periodically during treatment in children and adolescents, given the risk of cardiac complications, including reports of sudden death at high doses. Other adverse effects include dry mouth, constipation, and tachycardia [31]. Imipramine may be particularly useful in patients with comorbid depression or anxiety and is generally prescribed at doses of 1–5 mg/kg/day, divided into two daily doses. In clinical practice, the authors prefer limiting doses to ≤ 2 mg/kg/day, since cardiovascular complications have been associated with higher doses. Careful monitoring with ECG—or more detailed cardiovascular evaluation with cardiology follow-up, when indicated—is essential [31].

Alpha-adrenergic agonists, such as clonidine and guanfacine (the latter not yet available in Brazil), show greater efficacy for impulsivity, hyperactivity, and aggression, while being less effective for inattention. They may be considered in patients with tics or insomnia, with a reported effect size of 0,61 [32]. These medications should not be used when inattention is the predominant symptom and are contraindicated in patients with a history of arrhythmias, syncope, or depression. Abrupt discontinuation carries a risk of rebound hypertension. In Brazil, clonidine is only available as an immediate-release formulation, requiring two or three daily doses, which increases the risk of its most frequent side effect, sedation. Titration typically begins at 0025 mg/day, with doses up to 0,2 mg/day [32]. Other side effects include dry mouth, hypotension, and, more rarely, psychiatric manifestations. Evidence of efficacy is strongest in patients with ADHD and Tourette's Syndrome, where clonidine has limited effects on inattention but is beneficial for hyperactivity and impulsivity.

Bupropion, an antidepressant with dopaminergic and noradrenergic activity, has demonstrated moderate efficacy in ADHD, though its effect size is significantly smaller than that of stimulants. The recommended dose is 2–6 mg/kg/day, up to a maximum of 250 mg in children (not FDA-approved for this age group) and 300–400 mg in adolescents. Reported side effects are generally mild and include insomnia, weight loss, anxiety, agitation, and dry mouth. However, bupropion carries a slightly higher risk than other antidepressants of lowering the seizure threshold, and it is contraindicated in patients with epilepsy or eating disorders. In adolescents and adults, its utility extends to patients with substance use disorders (including nicotine dependence) and mood disorders [33].

Ultimately, the choice of medication should be individualized, considering the onset and duration of action, the pharmacological formulation, and the side-effect profile. While stimulants remain the first-line treatment for ADHD, initiation with a non-stimulant may be considered in patients with comorbidities such as anxiety, tics, sleep disturbances, mood dysregulation, or substance use disorders. Importantly, none of these comorbidities contraindicate stimulant use, and robust evidence indicates that stimulants can be prescribed effectively and safely in these contexts [34].

Table 3 presents the predominant side effects of the main medications that may be used in ADHD therapy.

**Table 3:** Predominant side effects of the main medications that may be used in ADHD therapy.

Medication	Most common	Less common	Rare
Methylphenidate	Insomnia, appetite loss, headache, abdominal pain	Irritability, anxiety, lethargy	Psychosis, arrhythmia
Lisdexamfetamine	Insomnia, appetite loss, headache, abdominal pain	Irritability, anxiety, lethargy	Psychosis, arrhythmia
Atomoxetine	Appetite loss, drowsiness, headache, abdominal pain	Nausea, vomiting, dizziness	—
Imipramine	Dry mouth, drowsiness	Appetite loss, urinary retention, tachycardia	Cardiac conduction abnormalities
Bupropion	Insomnia, headache, seizures	Allergy	—
Clonidine	Drowsiness, hypotension	Nausea, abdominal pain, mucosal dryness, photophobia	Arrhythmia

At present, there are no well-established clinical or biological markers to predict treatment response in ADHD. As a result, selecting the most effective medication typically depends on a guided process of trial and error. Optimization of stimulant therapy typically requires weekly titration, and determining the ideal dose may take several weeks. Dose titration to the maximum tolerated level is generally recommended to maximize efficacy.

Following the initiation of medication, frequent follow-up and careful dose titration are essential to maximize therapeutic efficacy and minimize adverse effects. In cases of stimulant-refractory ADHD, it is essential to investigate the underlying causes of non-response, which may include inadequate dose, poor adherence, adverse effects limiting dose escalation, or comorbidities mimicking or exacerbating ADHD symptoms.

With long-term use at higher doses, stimulants are associated with a small but significant increase in the risk of hypertension and arterial disease in children and adults, though more research is needed to clarify these associations [35].

There are currently no definitive guidelines on the optimal duration of pharmacological treatment or on when and how discontinuation should be attempted in ADHD. Evidence from discontinuation trials suggests that treatment benefits persist beyond the first two years, but most guidelines recommend annual reassessment of efficacy and tolerability [24].

The practice of “drug holidays”, particularly weekend breaks from stimulant use, remains debated. Arguments include:

• Against weekend breaks: ADHD symptoms affect not only academic performance but also family and social functioning, which extend into weekends. Moreover, academic demands often continue outside school days.
• In favor of weekend breaks: In selected patients where the primary treatment goal is academic performance, or when tolerability is a concern (e.g., appetite suppression, insomnia), weekend breaks may reduce side effects. Some clinicians also advocate planned drug holidays (weekends or vacations) to address concerns regarding growth delay, which tend to be small and reversible [36].

### Non-pharmacological treatments

While pharmacological therapy substantially improves core ADHD symptoms, it is often insufficient as a standalone approach. Optimal management requires an individualized, multimodal strategy that combines medication with psychosocial, educational, and behavioral interventions.

Psychosocial support includes psychoeducation for families and caregivers, behavioral management training, and guidance for daily routines. Educational interventions, such as psychopedagogical strategies, classroom accommodations, reinforcement of attention and self-regulation skills, and tailored assessment modifications, are crucial to improve academic performance, motivation, and self-esteem. Early interventions, particularly in children aged 3–5 years, are primarily non-pharmacological and play a pivotal role in shaping long-term outcomes [27].

Multidisciplinary involvement is often necessary. Psychologists provide cognitive-behavioral therapy and family guidance, speech-language therapists address communication difficulties, occupational therapists and physiotherapists support functional skills, and educational psychologists optimize learning strategies. The selection of professionals depends on individual patient needs. Family and school engagement is a key determinant of treatment success.

The pediatrician increasingly plays a central role beyond developmental assessments, facilitating early detection, suggesting the diagnostic hypothesis, providing education on ADHD and its prognosis, coordinating referrals, and monitoring treatment adherence and clinical evolution. By serving as the central point of communication among family, school, and the multidisciplinary team, the pediatrician ensures integrated, continuous care and prevents fragmentation of management.

Emerging interventions, such as neurofeedback and computer-based cognitive training, are under investigation, but current evidence is insufficient to recommend routine use. Further research is needed to determine their efficacy, optimal protocols, and long-term impact on ADHD outcomes.

## Prognosis

Longitudinal studies and neuroimaging research suggest that a proportion of individuals with ADHD show clinical improvement as brain maturation progresses, particularly during adolescence and early adulthood. Nevertheless, most patients remain symptomatic, with an estimated 60–70 % of those diagnosed in childhood continuing to experience clinically significant symptoms into adult life, although the severity and specific symptom profile may evolve over time [3]. The main predictors of symptom persistence in adulthood, according to a study of 140 individuals with ADHD, were higher baseline symptom severity, presence of psychiatric comorbidities, and a family history of psychiatric disorders [37]. Similarly, a prospective Brazilian cohort of 393 participants found that persistence into adulthood was also linked to more severe symptoms at onset and the presence of comorbidities such as oppositional defiant disorder or social phobia at baseline [38].

ADHD is associated with significant psychosocial impairments across multiple domains, including academic underachievement, difficulties in peer and family relationships, and challenges in daily activities, all of which directly affect quality of life as consistently reported in the literature. Individuals with ADHD show higher rates of school failure, are more likely to experience family and peer conflicts, and are at increased risk of rejection or bullying in the classroom. Children with ADHD exhibit lower levels of social skills, impaired social cognition, and have fewer and less stable friendships.

Family relationships are frequently characterized by increased conflict, negative interactions, and overall family burden. Parent-child discord is common, and the chronic nature of ADHD symptoms can lead to persistent family stress and increased healthcare costs. The familial challenges often persist into adulthood, manifesting as difficulties in marital and other interpersonal relationships. These adversities, when combined, may lead to diminished self-esteem and feelings of worthlessness, thereby increasing vulnerability to other neuropsychiatric disorders later in life [39].

ADHD has been further associated with increased risk of traffic accidents, substance misuse, occupational impairment, obesity, and suicide attempts. Elevated mortality rates have also been documented among patients with ADHD, as reported by Dalsgaard and colleagues, with accidents representing the leading cause of death. Notably, the highest mortality rates were observed among those diagnosed only in adulthood [40].

Encouragingly, current evidence shows that treatment of ADHD substantially improves quality of life, academic performance, accident risk, and emergency admissions due to trauma, and appears to confer protective effects against substance use disorders and suicide risk. Although treated patients generally achieve better outcomes than untreated individuals, complete symptom remission is rarely attained.

## Conclusion

In summary, ADHD is a prevalent and potentially disabling condition, carrying a substantial risk of symptom persistence and functional impairment into adulthood. Outcomes, however, are heterogeneous and influenced by factors such as symptom severity, co-occurring conditions, family and environmental context, and the duration of treatment. Early and continuous intervention can significantly improve long-term prognosis. The pediatrician, due to their privileged position for longitudinal follow-up, plays a central role in the detection, guidance, and coordination of multidisciplinary care, contributing to the child’s healthy development and full inclusion in their life contexts. Ongoing monitoring for residual symptoms and comorbidities remains essential to optimize outcomes.

## Data availability statement

The data that support the findings of this study are available from the corresponding author.

## Conflicts of interest

Erasmo Barbante Casella: Has given lectures over the past five years for: Adium, Apsen, EMS, Mantecorp, Takeda, and TEVA. He also served on the advisory committee for EMS and Mantecorp during this period. Beatriz Borba Casella: Has given lectures over the past five years for Apsen and EMS.
